# In-hospital hip fracture mortality score: predicting mortality after surgery for proximal femoral fracture in older patients

**DOI:** 10.1080/07853890.2026.2700053

**Published:** 2026-07-28

**Authors:** Erica Blenda da Silva, Fernanda Yuri Yuamoto, Mauricio Wesley Perroud Junior, Rodrigo Gonçalves Pagnano, Guilherme Grisi Mouraria

**Affiliations:** a Department of Orthopedics, Rheumatology and Traumatology—School of Medical Sciences, State University of Campinas (UNICAMP), Campinas, Sao Paulo, Brazil; b Department of Internal Medicine—School of Medical Sciences, State University of Campinas (UNICAMP), Campinas, Sao Paulo, Brazil

**Keywords:** Frail elderly, hip fractures, hospital mortality, risk factors, risk assessment

## Abstract

**Background/Objective/Introduction:**

Hip fractures in older patients are associated with substantial morbidity and in-hospital mortality. Despite the clinical importance of this condition, few tools specifically estimate in-hospital mortality or account for clinical deterioration during hospitalization, particularly in middle-income settings. This study aimed to develop a dynamic prognostic score to estimate the risk of in-hospital mortality among older patients undergoing surgical treatment for hip fractures.

**Materials and Methods:**

A retrospective cohort study was conducted with 1,509 patients aged 60 years and older treated at a Brazilian trauma centre between 2013 and 2023. Demographic, clinical, orthopaedic, laboratory and in-hospital evolution variables were analysed. Independent predictors of mortality were identified using binary logistic regression. A prognostic score was constructed using weights derived from the magnitude of association (odds ratios) observed in the logistic regression. Model accuracy was assessed by the area under the ROC curve (AUC). Patients were stratified into four in-hospital death risk categories: low, moderate, high, and very high.

**Results:**

The in-hospital mortality rate was 3.7%. Independent predictors included dementia, acute myocardial infarction, heart failure, and abnormalities in creatinine, potassium, and INR. In-hospital events including pulmonary thromboembolism, need for blood transfusion, and postoperative ICU admission were also strongly associated with death. The final score showed good discriminative performance with an AUC of 0.819. Predicted in-hospital mortality increased progressively across the risk groups, from 3%, among low-risk patients to > 20% among very-high-risk patients.

**Conclusions:**

The In-Hospital Hip Fracture Mortality Score demonstrated good predictive ability for in-hospital mortality in older surgically treated patients with hip fractures. However, it should be considered a preliminary hospital-based tool, and external validation in independent cohorts is needed before routine clinical implementation

## KEY MESSAGES

1. **A new prognostic tool:** The In-Hospital Hip Fracture Mortality Score is a good-performing tool (AUC 0.819) specifically developed to predict in-hospital mortality for older patients with hip fractures, with potential relevance to middle-income settings.
2. **Dynamic risk markers:** Blood transfusion and postoperative intensive care unit (ICU) admission were identified as important dynamic markers of physiological deterioration, significantly increasing the risk of in-hospital death.
3. **Clinical application:** This score supports dynamic risk reassessment during hospitalization into four categories, enabling clinicians to tailor care plans, allocate resources efficiently, and improve communication with family members regarding postoperative in-hospital risk. External validation is required before routine clinical use.

## Introduction

Hip fractures represent a growing public health concern worldwide. With population aging and increasing life expectancy, the incidence of osteoporotic hip fractures is expected to rise substantially in the coming decades [1,2]. Among frailty-related conditions affecting older adults, hip fractures are associated with significant morbidity, functional loss, and high mortality [3,4].

Several factors are associated with HF occurrence in older adults. Advanced age, female sex, and low socioeconomic status feature among the main population determinants, as well as the presence of comorbidities such as dementia, heart failure, diabetes mellitus, and chronic obstructive pulmonary disease (COPD) [5,6]. Osteoporosis is a key factor and is influenced by low serum vitamin D levels, sedentary lifestyle, and chronic use of medications like corticosteroids, antiseizure medications and selective serotonin reuptake inhibitors [7–10]. Environmental factors that predispose to falls and functional factors such as cognitive deficit and sarcopenia also increase their vulnerability [11].

Complications after HF surgery are frequent and often severe. Prolonged immobility and delays to surgery may favor the occurrence of pneumonia, venous thromboembolism, delirium, and urinary infections, negatively impacting patient prognosis [12,13]. Mortality rates after hip fracture remain substantial, with reports of approximately 5–10% at 30 days and up to 30% at 1 year, despite surgical treatment [12–15]. In-hospital mortality also remains clinically relevant, particularly in frail patients with multiple comorbidities or postoperative complications [14–17]. Reports from other international cohorts indicate in-hospital mortality rates between 1.5% and 11.4% [18–21]. Need for blood transfusion, prolonged surgical time, indication for recovery in the intensive care unit (ICU), and sustained functional loss after hospital discharge are frequent events associated with worse clinical outcomes [22–24].

Some prognostic scores have been investigated to estimate the risk of mortality in elderly patients with HF. The Nottingham Hip Fracture Score (NHFS), although widely validated across multiple international cohorts, was primarily developed to predict 30-day mortality and relies on variables that are often difficult to obtain [25–27]. Other models, such as POSSUM and its orthopedic adaptations, have been applied in this population, as reported by Yang et al. but have shown limited predictive performance for in-hospital mortality [28]. Similarly, the Charlson Comorbidity Index has been evaluated in this setting. However, its use has primarily been associated with medium- and long-term outcomes rather than in-hospital events [29,30].

Few models specifically address in-hospital mortality risk after hip fracture surgery, and most have been validated only in high-income countries [18,20]. In addition, most existing tools are based predominantly on baseline or preoperative characteristics and do not account for clinical deterioration or major events occurring during hospitalization. Their applicability to middle-income settings may therefore be limited.

In this context, there is a need for a simple and objective prognostic tool capable of estimating in-hospital mortality while also incorporating clinical evolution during hospitalization. A model that combines baseline characteristics with in-hospital events may provide a more realistic estimate of short-term mortality risk and support ongoing risk reassessment, resource planning, and communication with families.

Therefore, this study aimed to develop a dynamic prognostic score based on readily available clinical and laboratory parameters, combined with relevant in-hospital events, to estimate in-hospital mortality after hip fracture surgery in older patients.

## Materials and methods

A retrospective cohort study was conducted based on medical record analysis of older patients with hip fractures admitted to a tertiary trauma referral center between January 2013 and December 2023.

Eligible cases were initially identified through institutional hospital databases using ICD-10 codes corresponding to proximal femoral fractures (S72.0; S72.1; S72.2). Patients younger than 60 years were excluded at this stage. Remaining exclusion criteria were assessed through individual medical record review.

Inclusion criteria comprised patients aged 60 years or older diagnosed with hip fractures who underwent surgical treatment. Patients aged > 60 years were included, as this cutoff is used in Brazil to define older adults according to national legislation [31]. In addition, recent World Society of Emergency Surgery guidelines acknowledge that definitions of geriatric trauma patients vary across studies and settings, with thresholds ranging from 60 to 65 years or more, reinforcing that cutoff should be interpreted according to the clinical and regional context [32].

Exclusion criteria included patients with COVID-19 infection, incomplete medical records, revision surgery during hospitalization, pathological fractures, and those lacking clinical conditions for surgical intervention. Patients who died before undergoing surgery were also excluded because the objective of this study was to evaluate risk factors for in-hospital mortality specifically among surgically treated patients, with the aim of developing a prognostic score. Including preoperative deaths could introduce heterogeneity and confound the interpretation of predictors related to surgical treatment and postoperative outcomes. Patients with COVID-19 infection were excluded, as COVID-19 could influence perioperative management and outcomes due to infection-control protocols, changes in care delivery, surgical delays, and disease-related effects. This criterion was applied to reduce confounding and improve the comparability of the study population. Pathological fractures were defined as fractures related to malignant bone disease or other underlying bone lesions documented in the medical record or imaging reports.

Demographic, clinical, orthopaedic, laboratory, medical history data, and comorbidities were obtained from the patients’ medical records at the time of admission. In-hospital variables, including need for blood transfusion, pulmonary thromboembolism and postoperative ICU admissions were recorded during the hospital stay. Table 1 summarizes these variables.

**Table 1.** Baseline characteristics and clinical data analyzed.

**Category**	**Data collected**
Demographics	Age, sex
Laboratory tests	Hematocrit, hemoglobin, leukocytes, INR, creatinine, sodium, potassium
Concomitant fractures	Distal radius fracture, proximal humerus fracture, other associated fractures
Medical history	Smoking, chronic obstructive pulmonary disease (COPD), systemic arterial hypertension (SAH), heart failure (HF), acute myocardial infarction (AMI), ischemic and hemorrhagic stroke, deep vein thrombosis (DVT), use of full anticoagulation, Alzheimer’s disease, Parkinson’s disease, cerebral palsy, dementia, diabetes mellitus, hypothyroidism
In-hospital outcomes	Need for blood transfusion, ICU admission, pulmonary thromboembolism (PTE)
Orthopedic data	Anatomical location of the fracture (femoral neck, intertrochanteric, subtrochanteric), type of surgery performed (osteosynthesis: cannulated screw, cephalomedullary nail, DHS*, DCS**; hip prosthesis: bipolar or total)
In-hospital data	Date of admission, date of surgery, date of discharge or death, cause of death

*Dynamic hip screw.

**Dynamic condylar screw.

Laboratory variables were obtained from admission blood tests and represented baseline physiological status. Continuous variables, including laboratory parameters such as creatinine, potassium and INR, were initially analysed as continuous variables in the logistic regression model. After the identification of independent predictors of in-hospital mortality, variables retained in the final model were converted into dichotomous variables for construction of the prognostic score. Laboratory variables were categorized based on clinically relevant thresholds according to institutional reference ranges (creatinine > 1.2 mg/dL, INR > 1.2, and potassium levels < 3.6 or > 5.2 mmol/L).

Candidate predictors were initially selected based on clinical relevance and evidence from the literature regarding factors potentially associated with in-hospital mortality after hip fracture. After this initial selection, multicollinearity among candidate predictors was assessed using variance inflation factor (VIF) and tolerance values. Only variables with a tolerance greater than 0.1 and a Variance Inflation Factor (VIF) lower than 10 were retained. All selected variables were included in a binary logistic regression model.

The discriminative capacity of the model was evaluated using the receiver operating characteristic (ROC) curve, with calculation of the area under the curve (AUC). Two scoring approaches were compared: one assigning equal weight (1 point) to each significant predictor and another assigning weights proportional to the odds ratio (OR) derived from logistic regression. The model with the highest AUC was selected as the final scoring system.

Risk categories were defined according to the observed relative risk of in-hospital death associated with each score value. After calculating the total score for each patient, the risk of death was evaluated across the range of score values and plotted graphically. Based on the progressive increase in mortality risk, the score was divided into four clinically interpretable risk strata: low, moderate, high, and very high risk. These categories were selected to reflect meaningful increases in the probability of in-hospital death and to facilitate clinical interpretation of the prognostic score.

The prognostic score was designed as a hospital-based risk stratification tool. Baseline demographic, clinical, comorbidity, and laboratory variables can support initial risk assessment at admission, while post-admission variables such as transfusion and ICU admission allow time-updated reassessment during hospitalization.

Comparisons between groups were performed using the Student’s t for continuous variables and Chi-square test or Fisher’s exact test for categorical variables, as appropriate.

Statistical analysis used the IBM^®^ SPSS^®^ Statistics software (version 31.0, IBM Corp., Armonk, NY, USA) considering a statistical significance level of 5% (*p* < 0.05).

This study was conducted in accordance with the ethical standards of the institutional research committee and the principles of the Declaration of Helsinki. Ethical approval was obtained prior to the initiation of the study from the Research Ethics Committee of the University of Campinas (UNICAMP), under CAAE number 77223123.6.0000.5404. As the study consisted exclusively of retrospective medical record analysis and no participants were under active follow-up, the requirement for written informed consent was waived by the Committee.

## Results

**Figure 1.**
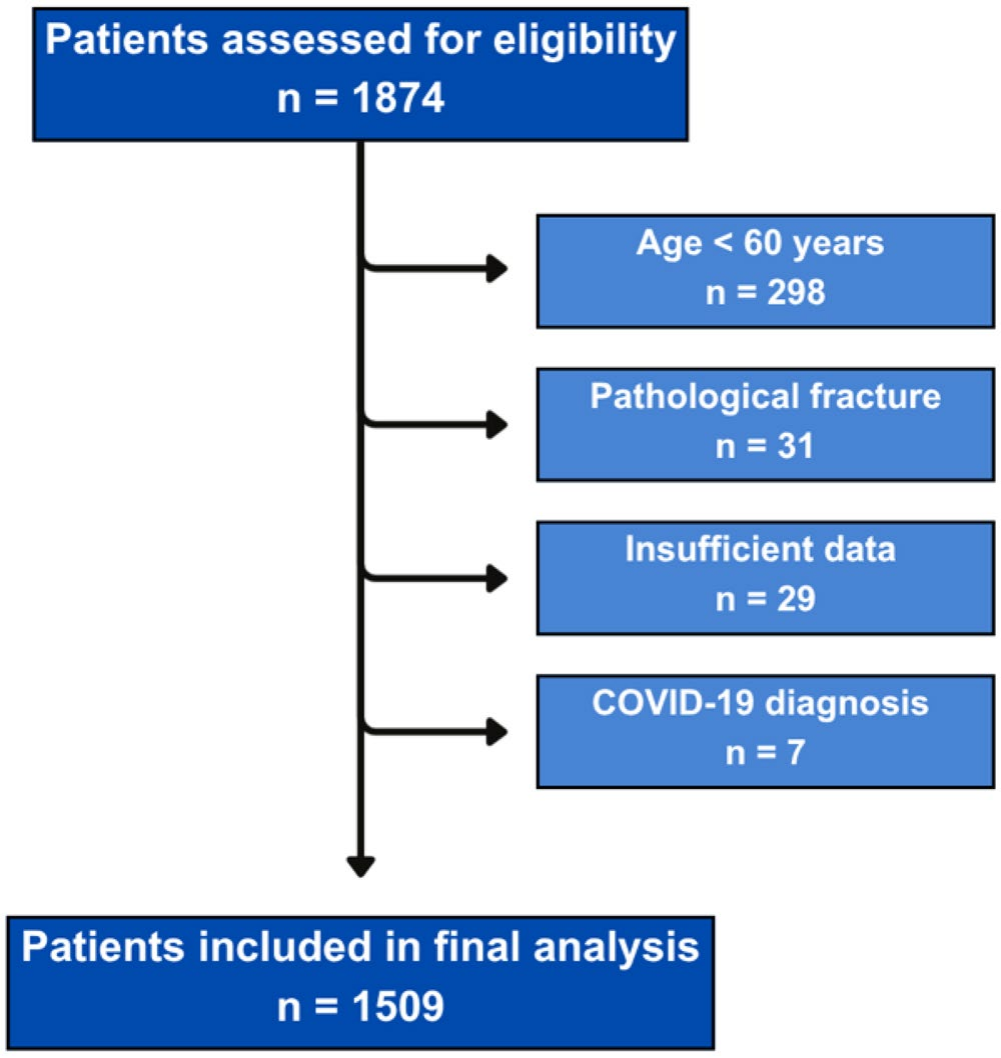
Flow diagram illustrating the inclusion and exclusion criteria and the final study population of patients with hip fracture.

Of the 1,874 patients with hip fracture initially identified, 1,509 were included after applying the inclusion and exclusion criteria. A case-flow diagram summarizing patient selection, exclusions, and the final study population is presented in Figure 1. The flowchart details the number of initially screened records, the number of patients excluded according to predefined criteria, and the final number of surgically treated patients included in the analysis.

The mean patient age was 78.15 ± 9.45 years, and most were female (67.3%). Anatomical distribution of the fractures showed a higher prevalence in the intertrochanteric region (59.6%), followed by femoral neck fractures (31.2%) and subtrochanteric fractures (9.1%). As for the type of surgical procedure, most patients underwent fixation with a cephalomedullary nail (31.7%) or dynamic hip screw (31.7%), while bipolar prosthesis was performed in 26% of the cases.

A high prevalence of systemic arterial hypertension (63.5%) and diabetes mellitus (28.3%) was observed among the study population. This initial analysis was performed to describe the clinical profile of the sample and to explore the distribution of comorbidities according to mortality status without establishing independent associations. Mortality was more frequent in patients with a history of heart failure, ischemic stroke, dementia, acute myocardial infarction, and chronic obstructive pulmonary disease (Table 2).

**Table 2.** Prevalence of comorbidities according to mortality status.

Comorbidity [n^o^ (%)]	Death n^o^	No Death n^o^	*P* Value
Systemic arterial hypertension [958 (63.4%)]	41	917	0.20*
Diabetes mellitus [427 (28.2%)]	20	407	0.29*
Stroke (ischemic stroke) [197 (13%)]	12	185	0.06*
Hypothyroidism [155 (10.2%)]	10	145	0.74*
Alzheimer’s disease [153 (10.1%)]	6	147	0.92*
Active smoking [144 (9.5 %)]	8	136	0.23*
Heart failure [132(8.7%)]	12	120	<0.001*
Dementia (unspecified) [ 89 (5.8%)]	7	82	0.03*
Acute myocardial infarction [ 82 (5.4%)]	7	75	0.02*
Chronic obstructive pulmonary disease [73 (4.8%)]	9	64	<0.001*
Cerebral palsy [2 (0.1%)]	0	2	0.77**
Parkinson’s disease [34 (2.2%)]	1	33	0.79**
Hemorrhagic stroke [5 (0.3%)]	0	5	0.65**

n^o^ = number of patients with the comorbidity; % = percentage of comorbidity among all patients; * = Chi-square test; ** Fisher′s exact Test.

The median length of hospital stay was 6 days (range, 1–74 days). The median time from admission to surgical treatment was 3 days (range, 1–41 days). Following surgery, the median time to discharge was 2 days (range, 1–90 days).

During hospitalization, 35% of the patients required postoperative intensive care and 18.6% received blood transfusion. The 57 in-hospital deaths corresponded to a mortality rate of 3.7%. There was no difference between sexes, with 36 deaths among women and 21 among men (*p* = 0.47). The mean age of patients who died was 77.8 years, which did not differ from that of patients who were discharged (78.1 years; *t* = 0.20; 95% CI = −2.3 to 2.7; *p* = 0.41).

Baseline characteristics stratified by mortality status, comparing survivors and non-survivors before adjustment, are presented in Supplementary Tables S1A and S1B.

Multicollinearity analysis demonstrated acceptable tolerance and Variance Inflation Factor (VIF) values for all variables included in the regression model (Supplementary Table S2).

A binary logistic regression model including the variables presented in Table 3 identified the following independent predictors of in-hospital mortality: elevated creatinine levels and changes in potassium and INR values on admission; previous comorbidities including heart failure, acute myocardial infarction, and dementia; and in-hospital factors including postoperative ICU admission, pulmonary thromboembolism, and blood transfusion.

**Table 3.** Influence of clinical variables on in-hospital mortality.

Parameter	OR	*p*-value	95%CI*
Postoperative ICU	8.4	<0.001	4.3 − 16.3
Pulmonary thromboembolism (PTE)	7.5	0.01	1.5 − 36.9
Blood transfusion	6.2	<0.001	3.6 − 10.6
Heart failure (HF)	2.9	<0.001	1.5 − 5.7
Previous acute myocardial infarction (AMI)	2.5	0.025	1.1 − 5.8
Dementia	2.3	0.040	1 − 5.3
Potassium < 3.6 or > 5.2	1.9	<0.001	1.3 − 2.8
INR > 1.2	1.8	0.005	1.2 − 2.9
Creatinine > 1.2	1.2	0.003	1 − 1.4

*CI = Confidence interval.

**Figure 2.**
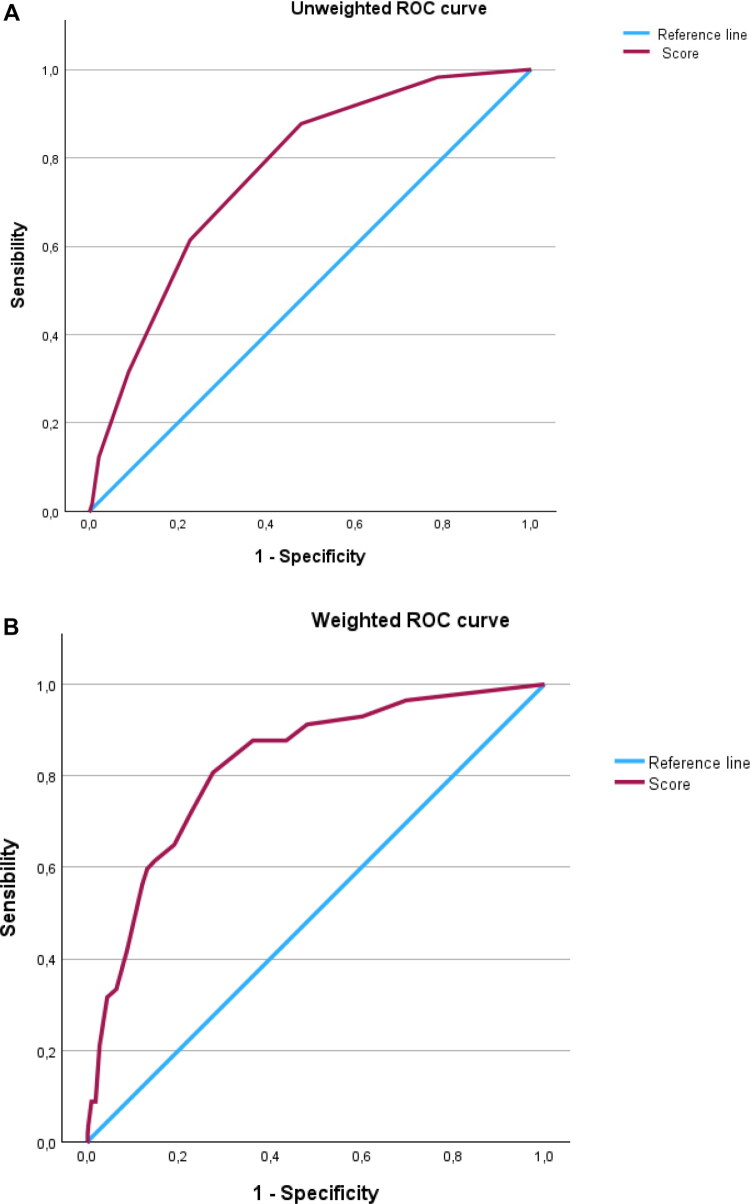
Comparison of ROC curves for mortality prediction. Figure 2A represents the unweighted model assigning one point to each variable, whereas Figure 2B represents the weighted model using weights proportional to the odds ratios.

ROC curve analysis was used to evaluate model accuracy and compare two scoring approaches: an unweighted model assigning one point to each significant variable associated with mortality and a weighted model using weights proportional to the odds ratios. For the unweighted model (Figure 2(A)), the optimal cutoff according to the Youden index was 2 points, yielding a sensitivity of 87.7%, specificity of 52.1%, positive predictive value of 6.7%, and negative predictive value of 99.1%. For the weighted model (Figure 2(B)), the optimal cutoff was 9 points, with a sensitivity of 80.7%, specificity of 72.5%, positive predictive value of 10.3%, and negative predictive value of 99.0%. The weighted model demonstrated better discriminatory performance than the unweighted model, with an AUC of 0.819 (95% CI = 0.765–0.873; *p* < 0.001) versus 0.789 (95% CI = 0.735–0.844; *p* < 0.001), respectively (Table 4).

**Table 4.** Comparison of area under the ROC curve with and without correction.

ROC Curve Type	Area Under the Curve (AUC)	*p*-value	Confidence Interval (95%)
Unweighted ROC curve	0.789	<0.001	(0.735–0.844)
Weighted ROC curve	0.819	<0.001	(0.765–0.873)

**Figure 3.**
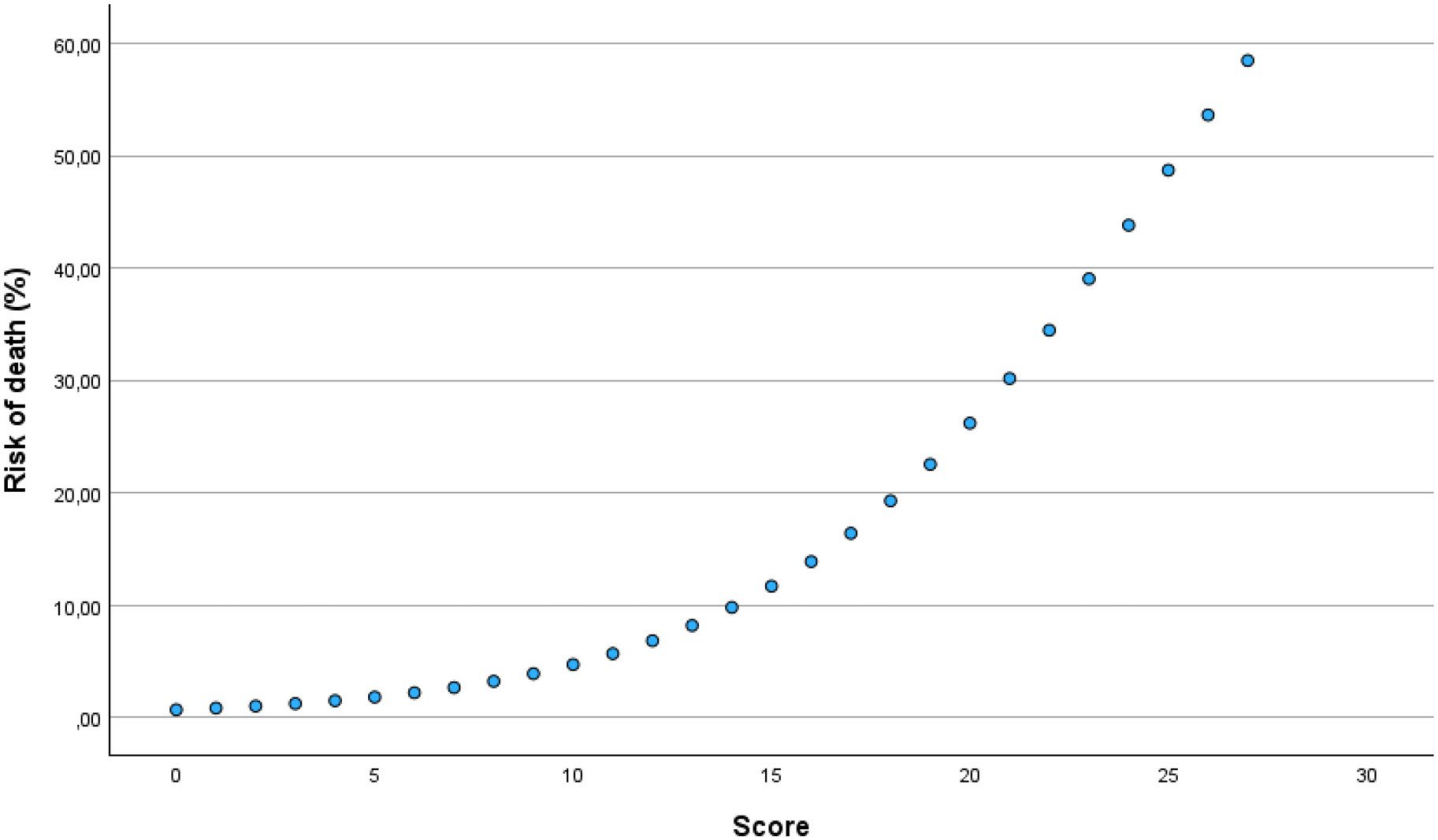
Association between score and risk of in-hospital mortality.

Based on the odds ratios, a prognostic score was constructed to estimate in-hospital mortality. Each significant variable was assigned a weight based on the magnitude of its OR derived from the multivariable logistic regression model, which was used to construct the score (Table 5). The total score was calculated for each patient, and the corresponding probability of mortality was estimated. Higher scores were associated with increased risk (Figure 3).

**Table 5.** Score attributed to the variables of the in-hospital mortality prognostic score.

Parameter	OR	Points awarded
Postoperative ICU admission	8.4	8
Pulmonary thromboembolism (PTE)	7.5	7
Need for blood transfusion	6.2	6
Heart Failure (HF)	2.9	3
Previous acute myocardial infarction (AMI)	2.5	2
Dementia	2.3	2
Potassium <3.6 or >5.2	1.9	2
INR >1.2	1.9	2
Creatinine >1.2	1.2	1

Figure 3 shows an exponential relationship between the score and the risk of mortality. The curve was stratified into four score ranges: low risk (mean mortality 1.48%), moderate risk (6.04%), high risk (15.3%), and very high risk (39.65%). The distribution of mortality according to score ranges is detailed in Table 6.

**Table 6.** Relationship between score, risk range, mean observed mortality and absolute number of deaths in each risk range.

Score	Risk range	Mean Observed Mortality (%)	n
0–7	Low (<3%)	1.48%	933
8–14	Moderate (3–10%)	6.04%	425
15–18	High (11–19%)	15.3%	120
≥19	Very High (>20%)	39.65%	31

The practical application of the final score is presented in Table 7.

**Table 7.** In-Hospital hip fracture mortality score.

Criteria	Score
**Previous comorbidities**	
Heart Failure	3
Acute Myocardial Infarction	2
Dementia	2
**Laboratory tests on admission**	
Potassium <3.6 or >5.2	2
INR >1.2	2
Creatinine >1.2	1
**Clinical evolution**	
Need for intensive care unit in the postoperative period	8
Pulmonary thromboembolism	7
Need for blood transfusion	6
Classification and risk of in-hospital mortality	
Final score	Risk of mortality
0–7	Low (<3%)
8–14	Moderate (3–10%)
15–18	High (11–19%)
≥ 19	Very High (>20%)

## Discussion

The in-hospital mortality rate was 3.7%, which is within the range reported in international cohorts (1.5% to 11.4%) [18–21]. These variations reflect differences in population profile, exclusion criteria and healthcare system factors. Although age and sex have been identified as predictors in previous studies [33], they were not retained as independent predictors in the final model for in-hospital mortality. This finding should not be interpreted as indicating that these variables are not clinically relevant. Rather, in the hospital setting, clinical conditions associated with advanced age and biological vulnerability may be more readily identified, monitored, and managed through perioperative care, laboratory correction, hemodynamic support, and intensive care when needed. In addition, the effects of age and sex may have been partially captured by variables more proximal to the in-hospital outcome, particularly postoperative factors such as ICU admission and blood transfusion, which may reflect clinical severity, frailty, perioperative complications, and reduced physiological reserve. Because these postoperative variables may lie on the caudal pathway between baseline patient characteristics and in-hospital mortality, this represents an important consideration when interpreting the model.

The main predictors identified in this study are consistent with findings from international studies on hip fracture mortality. Cohorts from Europe, United States and Brazil have shown that comorbidity burden, cardiac disease, renal dysfunction, and postoperative complications are key determinants of in-hospital mortality, as observed in our study as well [18,21,34]. Unlike traditional models based exclusively on baseline characteristics, the present score combined admission variables with relevant in-hospital events, allowing dynamic reassessment of mortality risk during hospitalization.

Independent associations between perioperative transfusion and increased mortality have been reported in large population-based cohorts, where adjusted analyses demonstrated higher 30- and 60-day mortality among transfused patients [35]. However, few studies specifically correlate in-hospital mortality with transfusion events. Additional evidence shows that transfusion is associated with postoperative increased complication rates, delirium and longer hospital stays in frail elderly patients [36,37]. Admission haemoglobin, although associated with higher mortality in some studies [34,38], was not independently associated with in-hospital mortality in our cohort. The absence of an independent association between admission haemoglobin and in-hospital mortality should be interpreted with caution. Although anaemia has been associated with worse outcomes after hip fracture, its effect in the present model may have been partially captured by blood transfusion, which was retained as a predictor. Because transfusion is often performed in response to low haemoglobin level, perioperative blood loss, or clinical instability, it may lie downstream of admission haemoglobin and act as a mediator between anaemia and mortality. Therefore, the inclusion of transfusion in the multivariate model may have attenuated the direct association between haemoglobin and mortality. This reinforces that the proposed score should be interpreted as a pragmatic tool for in-hospital mortality rather than as a causal model.

In binary logistic regression, need for blood transfusion (OR = 6.2; 95% CI 3.6–10.6, *p* < 0.001) and postoperative ICU (OR = 8.4; 95% CI 4.3–16.3, *p* < 0.001) were among the predictors with the greatest impact on in-hospital mortality. Based on these results, both blood transfusion and the need for postoperative ICU should be understood as dynamic markers of physiological decompensation and not only as care variables. Their requirement may indicate the transition from an initially stable patient to a more severe clinical course.

The inclusion of variables such as blood transfusion and postoperative ICU admission requires careful interpretation from a temporal perspective. These variables are not purely baseline predictors available at hospital admission; rather they are dynamic markers that reflect clinical evolution during the perioperative and postoperative period. Therefore, the proposed score should not be interpreted exclusively as a baseline admission-only model. Instead, it is best understood as a pragmatic hospital-based prognostic tool that can be applied in two complementary moments: first, at the admission using demographic, clinical, comorbidity and laboratory information available early in hospitalization, and subsequently updated during the hospital course when dynamic events such as transfusion requirement, thromboembolism and ICU admission occur.

Several prognostic scores have been developed to estimate the risk of mortality in older patients with hip fractures, among which the Nottingham Hip Fracture Score (NHFS), widely validated in different clinical contexts [25,26], including a Brazilian cohort [27]. It incorporates variables such as age, sex, institutionalization, comorbidities, haemoglobin level, and mental status, and is especially useful in predicting 30-day mortality [39]. Despite the relevance of this tool, few scores focus on in-hospital mortality, limiting the ability to estimate immediate outcomes during hospitalization.

Among the few existing models, the score proposed by Endo et al. (2018), derived from a large US hospital database, is notable and includes variables such as age, sex, congestive heart failure, and fluid and electrolyte disorders [18]. Similarly, Sanz-Reig et al. (2018) developed a score to assess in-hospital mortality in a Spanish cohort [20]. Consistent with our findings, heart failure was also identified as an independent predictor of in-hospital mortality in these studies. However, none of these existing scores are validated for use in populations of middle-income countries. The distinguishing feature of the tool presented here is the integration of baseline laboratory variables with acute in-hospital clinical events, which may better reflect evolving physiological severity. These factors can be interpreted as signs of clinical deterioration, allowing for continuous reassessment of risk based on patient course. Furthermore, the use of primary and homogeneous clinical data collected directly from medical records at a single referral center likely contributed to the observed performance of the model, minimizing interference related to care variability and data omission common in secondary databases.

Assigning weights proportional to the odds ratios improved performance (AUC from 0.765 to 0.819) and highlighted the cumulative impact of risk. Stratification into risk categories (low, moderate, high and very high) may facilitate clinical application by supporting graded decision-making, from closer monitoring to early discussions of care goals in higher-risk patients. As limitations, the retrospective study design should be acknowledged, as it relies on secondary records and is subject to information bias. In addition, the single-center sample may limit the generalizability of the findings. Because postoperative variables were included, the score is intended for dynamic risk reassessment during hospitalization. Although the proposed score showed an association with in-hospital mortality in the study cohort, external validation in an independent cohort was not available in the present study. Therefore, the score should be interpreted as a preliminary prognostic tool and requires validation in independent populations before broader clinical implementation. Future studies should assess its calibration, discrimination, and clinical utility in external cohorts.

By incorporating comorbidities, admission laboratory data, and dynamic markers of clinical decompensation, the score may support ongoing risk reassessment throughout hospitalization. In this context, baseline variables contribute to initial risk stratification, whereas dynamic variables refine risk estimation as patients evolve. This approach reflects real-world inpatient care, in which prognosis is continuously reassessed after surgery and postoperative monitoring. However, because these dynamic variables may occur after admission and may lie on the causal pathway between baseline status, perioperative complications, and mortality the score should be interpreted as a time-updated prognostic tool rather than as strictly baseline causal model.

Prospective, multicentre studies are needed for external model validation and analysis of impact on clinical practice.

## Conclusion

The In-Hospital Hip Fracture Mortality Score showed good predictive performance for in-hospital mortality among older patients undergoing surgical treatment for hip fractures. However, the score should be considered a preliminary hospital-based prognostic tool. Although it may support risk stratification within the study cohort, external validation in independent populations is required before routine clinical use.

## Supplementary Material

TFSupplementaryTableS2.docx

TFSupplementary table 1.docx

## Data Availability

The anonymized dataset supporting the findings of this study is publicly available in REDU, at https://doi.org/10.25824/redu/3ZNC3T
