# Supplementary material for: In-hospital hip fracture mortality score: predicting mortality after surgery for proximal femoral fracture in older patients
Source: Ann Med. 2026 Jul 28;58(1):2700053. doi: 10.1080/07853890.2026.2700053 (PMC13421109; doi:10.1080/07853890.2026.2700053)
Supplement: TFSupplementaryTableS2.docx [file IANN_A_2700053_SM0105.docx]

**Supplementary Table S2.** Multicollinearity test of variables included in the regression model

Coefficients^a^

| Variable | Beta  (standardized coefficient) | t | Sig. | Tolerance | VIF |
| --- | --- | --- | --- | --- | --- |
| Age | -0.038 | -1.210 | 0.227 | 0.743 | 1.346 |
| Sex | -0.003 | -0.117 | 0.907 | 0.844 | 1.185 |
| Short Gamma Nail | -1.355 | -5.626 | <0.001 | 0.013 | 79.956 |
| Long Gamma Nail | -0.741 | -5.965 | <0.001 | 0.047 | 21.265 |
| Cannulated Screw Fixation (Femoral Neck) | -0.426 | -5.011 | <0.001 | 0.100 | 9.967 |
| Bipolar Prosthesis | -1.160 | -5.137 | <0.001 | 0.014 | 70.257 |
| Dynamic Hip Screw (DHS) | -1.239 | -5.483 | <0.001 | 0.014 | 70.422 |
| Dynamic Condylar Screw (DCS) | -0.417 | -5.371 | <0.001 | 0.121 | 8.297 |
| Total Hip Arthroplasty | -0.128 | -4.059 | <0.001 | 0.732 | 1.367 |
| Femoral Neck Fracture | 0.258 | 0.592 | 0.554 | 0.004 | 261.384 |
| Intertrochanteric Fracture | 0.329 | 0.727 | 0.468 | 0.004 | 283.042 |
| Subtrochanteric Fracture | 0.338 | 1.299 | 0.194 | 0.011 | 93.526 |
| Concomitant Fractures | 0.036 | 1.307 | 0.192 | 0.950 | 1.053 |
| Time from Trauma to Surgery | -0.005 | -0.173 | 0.863 | 0.770 | 1.299 |
| Time from Admission to Surgery | 0.009 | 0.283 | 0.777 | 0.701 | 1.427 |
| Time from Surgery to Discharge/Death | 0.310 | 10.456 | <0.001 | 0.823 | 1.214 |
| Hypertension | -0.005 | -0.185 | 0.853 | 0.825 | 1.212 |
| Heart Failure | 0.009 | 0.314 | 0.754 | 0.865 | 1.157 |
| Diabetes Mellitus | -0.002 | -0.073 | 0.942 | 0.886 | 1.129 |
| Hypothyroidism | 0.006 | 0.212 | 0.832 | 0.951 | 1.052 |
| Alzheimer's Disease | -0.005 | -0.179 | 0.858 | 0.942 | 1.062 |
| Parkinson's Disease | 0.010 | 0.372 | 0.710 | 0.965 | 1.036 |
| Previous Hemorrhagic Stroke | -0.007 | -0.267 | 0.789 | 0.981 | 1.019 |
| Cerebral Palsy | -0.005 | -0.195 | 0.845 | 0.979 | 1.022 |
| Dementia | 0.011 | 0.399 | 0.690 | 0.949 | 1.054 |
| Chronic Obstructive Pulmonary Disease | 0.000 | -0.002 | 0.999 | 0.830 | 1.204 |
| Previous Myocardial Infarction | -0.003 | -0.095 | 0.924 | 0.897 | 1.115 |
| Previous Stroke | 0.045 | 1.586 | 0.113 | 0.913 | 1.095 |
| Previously Bedridden | 0.034 | 1.223 | 0.221 | 0.967 | 1.035 |
| Smoking | 0.030 | 1.014 | 0.311 | 0.805 | 1.243 |
| In-hospital Pulmonary Embolism | 0.010 | 0.372 | 0.710 | 0.949 | 1.054 |
| Deep Vein Thrombosis | -0.030 | -1.068 | 0.286 | 0.902 | 1.108 |
| Therapeutic Anticoagulation | 0.011 | 0.369 | 0.712 | 0.760 | 1.317 |
| Postoperative ICU Stay | 0.056 | 1.757 | 0.079 | 0.724 | 1.381 |
| Thromboprophylaxis from Day 1 | 0.028 | 0.992 | 0.321 | 0.933 | 1.072 |
| Serum Creatinine | 0.006 | 0.207 | 0.836 | 0.748 | 1.337 |
| Serum Sodium (Na) | -0.029 | -0.982 | 0.326 | 0.813 | 1.230 |
| Serum Potassium (K) | 0.014 | 0.435 | 0.664 | 0.750 | 1.334 |
| INR | -0.024 | -0.802 | 0.423 | 0.790 | 1.265 |
| Hemoglobin (Hb) | -0.064 | -1.898 | 0.058 | 0.633 | 1.581 |
| White Blood Cell Count | 0.013 | 0.460 | 0.646 | 0.921 | 1.085 |
| In-hospital Transfusion | 0.063 | 1.931 | 0.054 | 0.682 | 1.466 |

1. Dependent variable: mortality
