# Supplementary material for: In-hospital hip fracture mortality score: predicting mortality after surgery for proximal femoral fracture in older patients
Source: Ann Med. 2026 Jul 28;58(1):2700053. doi: 10.1080/07853890.2026.2700053 (PMC13421109; doi:10.1080/07853890.2026.2700053)
Supplement: TFSupplementary table 1.docx [file IANN_A_2700053_SM0104.docx]

**Supplementary Table 1A.** Comparation of admission laboratory parameters between survivors and non survivors

| **Variable (mg/dl)**  **(Mean+SD)** | **Survivors**  **(Mean+SD)** | **No-survivors**  **(Mean+SD)** | **P Value*** |
| --- | --- | --- | --- |
| Creatinine (1.21+0,93) | 1.61+1.08 | 1.19+0.92 | <0.001 |
| Sodium (137.86+3.91) | 138.05+4.47 | 137.85+3.89 | 0.43 |
| Potassium (4.19+0.59) | 4.45+0.72 | 4.19+0.58 | 0.002 |
| INR (1.09+0.29) | 1.22+0.45 | 1.08+0.28 | 0.002 |
| Hemoglobin (11.40+1.96) | 10.46+2.1 | 11.44+1.94 | 0.001 |

SD = standard deviation; * t student test

**Supplementary Table 1B.** Comparison of orthopedic characteristics between survivors and non-survivors

| **Variable**  **[n (%)]** | **Survivors**  **n^o^** | **No-survivors**  **n^o^** | **P -Value** |
| --- | --- | --- | --- |
| **Surgery procedure** Cephalomedullary nail (short) [478 (31.7%)] | 15 | 643 | 0.37* |
| Cephalomedullary nail (short) [87 (5.8%)] | 5 | 82 | 0.37** |
| Cannulated screw [34 (2.3%)] | 0 | 34 | 0.63** |
| Hip prosthesis [393 (26%)] | 16 | 377 | 0.71* |
| Dynamic hip screw [478 (31.7%)] | 17 | 461 | 0.75* |
| Dynamic condylar screw [36 (2.4 %)] | 2 | 34 | 0.64** |
| **Anatomical location’s fracture**  Femoral neck [471 (31.2%)] | 18 | 453 | 0,95* |
| Intertrochanteric [900 (59.6%)] | 28 | 872 | 0.09* |
| Subtrochanteric [137 (9.1%)] | 11 | 126 | 0.006* |
| Concomitant Fracture [45 (3.0%)] | 2 | 43 | 0.38** |

n^o^ = number of patients with the comorbidity; % = percentage of comorbidity among all patients; * = Chi-square test; ** Fisher´s exact test
